# Longitudinal tracking of single live cancer cells to understand cell cycle effects of the nuclear export inhibitor, selinexor

**DOI:** 10.1038/srep14391

**Published:** 2015-09-24

**Authors:** Joshua M. Marcus, Russell T. Burke, John A. DeSisto, Yosef Landesman, James D. Orth

**Affiliations:** 1 Department of Molecular, Cellular, and Developmental Biology, GOLD A240B, 347 UCB, University of Colorado—Boulder, Boulder, CO 80309 USA; 2 Karyopharm Therapeutics, Inc., 85 Wells Ave., Newton, MA 02459.

## Abstract

Longitudinal tracking is a powerful approach to understand the biology of single cells. In cancer therapy, outcome is determined at the molecular and cellular scale, yet relationships between cellular response and cell fate are often unknown. The selective inhibitor of nuclear export, selinexor, is in development for the treatment of various cancers. Selinexor covalently binds exportin-1, causing nuclear sequestration of cargo proteins, including key regulators of the cell cycle and apoptosis. The cell cycle effects of selinexor and the relationships between cell cycle effects and cell fates, has not been described for individual cells. Using fluorescent cell cycle indicators we report the majority of cell death after selinexor treatment occurs from a protracted G1-phase and early S-phase. G1- or early S-phase treated cells show the strongest response and either die or arrest, while those treated in late S- or G2-phase progress to mitosis and divide. Importantly, the progeny of cell divisions also die or arrest, mostly in the next G1-phase. Cells that survive selinexor are negative for multiple proliferation biomarkers, indicating a penetrant, arrested state. Selinexor acts quickly, shows strong cell cycle selectivity, and is highly effective at arresting cell growth and inducing death in cancer-derived cells.

Anti-cancer responses to small molecule drugs or natural products are determined on the molecular and cellular scale. Understanding cell responses and fates following treatment using population average assays (e.g. immunoblotting), masks cell-cell variability and differences in timing, and discounts transient and rare responses. To more completely understand the complexity of drug response we must track molecular responses and cell fate choices simultaneously in individual cells in real time. The use of long-term longitudinal approaches to follow a given single cell or a cell population is a less common but very powerful approach that allows for the direct study of molecular response pathways, different phenotypes (e.g. cell death or cell division), observation of cell-to-cell variability within a population, and how these factors contribute to population response dynamics^1^,^2^,^3^.

Targeting the cell cycle is a common rationale for the application and development of anti-neoplastic therapies, yet cell cycle specificity in targeting, observed effects on cell cycle progression, and cell cycle-associated cell death in single cells remain enigmatic. To directly monitor cell cycle progression in live cells we developed a human HT1080 fibrosarcoma-derived cell line that stably expresses the f luorescent ubiquitin cell cycle indicators (FUCCI)^4^,^5^. FUCCI cells become red in G1-phase and upon transition into S-phase show diminishing red fluorescence and increasing green fluorescence, resulting in orange to yellow transition in early S-phase, with a transition to entirely green in late S-phase. Cells remain green through G2-phase and mitosis, where upon anaphase the green probe is degraded. A direct monitoring approach allows for the observation of cell cycle arrest, but also progression defects, in which stage cells die, the timing and variability of events, the state of surviving cells and the relationship between cell cycle status when treated and fate decision—all in a single experiment. Further, time-lapse microscopy is a direct, longitudinal approach where an individual cell’s progression and ultimate fate in response to an agonist can be directly observed—not inferred—and population response dynamics can be studied, for example using survival curves^1^,^6^.

We followed individual cell responses and fates to different well-established cell cycle-targeted drugs, and the selective inhibitor of nuclear export (SINE) drug, selinexor. Selinexor binds covalently to the nuclear export protein exportin-1 (XPO1) at cysteine 528, resulting in blocked nuclear export and nuclear sequestration and accumulation of cargo proteins, including p53, pRB, p21^Cip1^ and p27^Kip1^ 7,8. Selinexor results in strong anti-cancer effects in a myriad of different cancer-derived cell lines and xenograft tumors^9^,^10^,^11^. However, single cell phenotypes, cell cycle specific effects, the timing of events, and relationships between cell cycle effects and death—if there are any—remain unknown. Through comparative analysis of the selinexor response against standard cell cycle manipulations, we conclude that most of the cell cycle arrest and death occurs in G1- or S-phase. Cells treated in G1- and early S-phase are more sensitive to selinexor, while those treated in G2-phase most often do not arrest or die and instead progress through cell division first and arrest or die in the subsequent G1- or S-phase. Longitudinal studies of individual cells is a powerful, unbiased approach to study drug response that can reveal both selectivity of action as well as profound variability in the timing and types of responses that occur within cells, allowing for a more complete understanding of drug response at the population level.

## Results

### FUCCI probes accurately report on cell cycle progression and arrest

**Figure 1:**
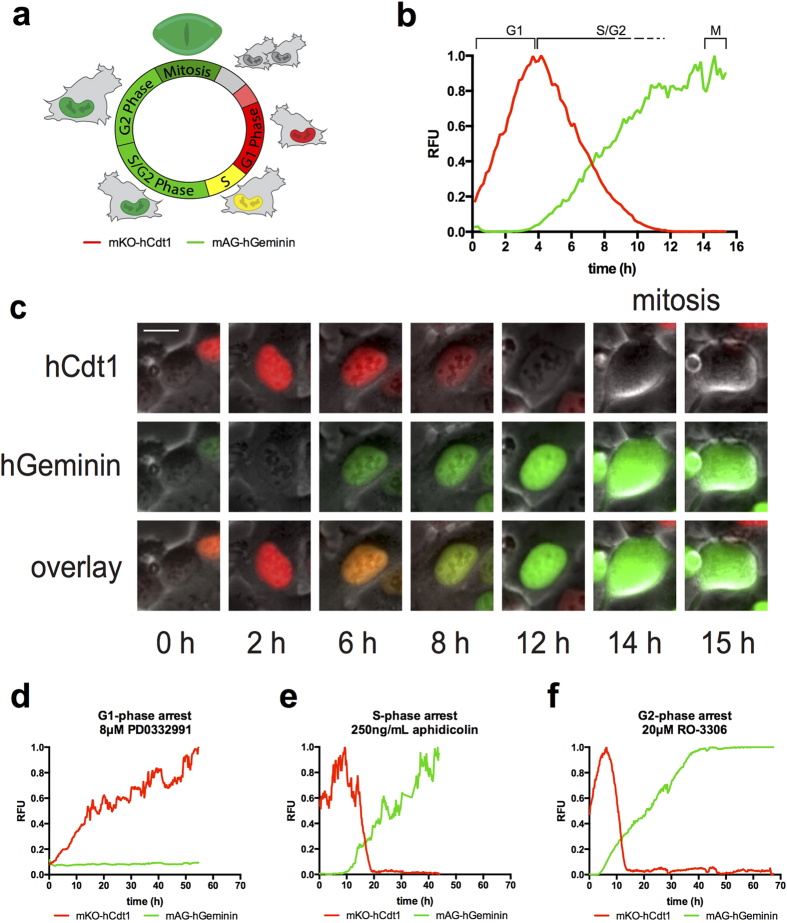
Characterization of cell cycle in HT1080 FUCCI cells. The FUCCI system (**a**) consists of two fluorescent polypeptides that are ubiquitylated and degraded by proteasomes in a cell cycle regulated manner. The red probe (mKO-hCdt1) increases in G1-phase and is degraded at the onset of S-phase. The green probe (mAG-hGeminin) increases at the onset of S-phase and is degraded upon anaphase of mitosis. (**b**,**c**) Untreated cells tracked from birth. As cells progress into S-phase, green signal accumulates and red signal continues to degrade while the green signal continues to increase. Absence of red signal corresponds to the transition to late S-phase. Cells remain green through late S- and G2-phase and enter mitosis after 14–18 h as indicated by observable breakdown of the nuclear envelope, and the green signal is rapidly degraded at anaphase onset (~15 h in this example). (**c**) FUCCI trace representing control cells measured from birth to mitosis. The average cell cycle time is ~16 h. Cells spend ~4–6 h in G1-phase, ~8–10 h in S/G2-phase, and ~1.0 h in mitosis. Bar is 10 μm. (**d**–**f**) Cell cycle arrest standards. (**d**) A representative FUCCI trace of cell born into the G1-phase arrestor, Cdk4/6 inhibitor, PD-0332991. Cells progress into G1-phase (red) and remain until the end of acquisition (60 h). (**e**) A representative FUCCI trace of cell treated with S-phase arrestor, DNA polymerase-α inhibitor, aphidicolin. Cells tracked from G1-phase (red), progress into S-phase with normal kinetics and become green. Cells remain green until the end of acquisition (45 h). (**f**) A representative FUCCI trace of cell treated with the G2-arrestor, Cdk1 inhibitor, 20 μM RO-3306. Cells tracked from G1-phase (red), progress normally into S-phase with normal kinetics and become green. Cells remain green until the end of acquisition (70 h). See Supplementary Fig. 1a–d online for FUCCI distribution over time. Supplementary videos S1, S2, S4, S5 online. Cell number tracked: untreated, 22, PD0332991, 19, aphidicolin, 24, RO-3306, 20.

Phase-contrast with fluorescent time-lapse microscopy was used to track growing FUCCI expressing cells; please see Fig. 1a for a graphical summary of the FUCCI sensors. FUCCI sensors consist of two fluorescent peptides that report on cell cycle stage^4^,^12^,^13^. Normal cells tracked from birth into G1-phase until the next cell division show an average 4–6 hours (h) of G1-phase (red) followed by approximately 6–8 h of S-phase (some red and some green), approximately 4 h of G2-phase (green), followed by mitosis; on average the cell cycle time is ~15 h (69 cells) (Fig. 1b,c, Supplementary video S1 online, please see Supplementary video legends file for details). Yellow cells are indicative of early S-phase^4^. Untreated cells show consistent FUCCI red, yellow, and green distribution until becoming confluent at approximately 16 h (Supplementary Fig. S1a online). We will refer to red cells as G1-phase, yellow as early S-phase and green as S/G2-phase throughout the text.

Prior to studying selinexor response we used well-characterized small molecules to validate the FUCCI sensors. We used two separate methods to arrest cells in a G1-state. The Cdk4/6 inhibitor PD0332991 acts rapidly on cells in early G1-phase and prevents their transition to S-phase^14^,^15^. Cells treated in G1-phase or born into PD0332991 (8 μM) early in the time-lapse remain red >90% of the time, for as long as 60 h (Fig. 1d, Supplementary video S2 online). Upon treatment, 37% of cells were in S/G2-phase. Most of these cells progress normally through mitosis, and arrest in the subsequent G1-phase. By 48 h, nearly 100% of cells are red (Supplementary Fig. S1b online). A small sub-population of cells (<10%) that were G1- or early S-phase when treated, transitioned to S/G2-phase, and shifted back to a G1-state (Supplementary video S3 online). This phenotype was observed previously with the Cdk4 selective inhibitor CAS 546102-60-7 (Calbiochem 219476)^13^. Because PD0332991 is a targeted small molecule that could possibly exert stress effects that perturb cell state, we also used serum deprivation with 0.2% FBS medium to promote a G1-phase state. By 72 h, cells are predominantly red (Supplementary Fig. S2 online). Each of these standard but independent G1-phase perturbations result in arrested red fluorescent cells, indicating strong G1-phase arrest.

Reversible, early S-phase synchronization (often termed G1/S synchrony) can be achieved via standard methods, including double-thymidine block or a single aphidicolin (DNA polymerase-α and δ inhibitor) treatment^16^. We used aphidicolin so we could observe the synchronization of the culture. Cells were treated with 250 ng/ml aphidicolin and followed using continuous time-lapse microscopy. Aphidicolin treated G1-phase cells progress from G1- into S-phase with kinetics similar to untreated cells, and essentially all arrest as green cells (Fig. 1e, Supplementary video S4 online); during aphidicolin treatment, cells arrest and remain only green. By 24 h, ~90% of cells are yellow or green, indicating strong S-phase arrest consistent with inhibition of DNA polymerases (Supplementary Fig. S1c online).

G2-phase arrest can be achieved using the reversible CDK1 inhibitor RO-3306^17^,^18^. Single cell longitudinal tracking upon RO-3306 treatment of non-synchronized FUCCI cells reveals that treated G1-phase cells progress with normal kinetics and arrest as completely green cells (Fig. 1f); we observe no loss of green signal or re-acquisition of red in RO-3306 (Supplementary video S5 online). Cells treated with RO-3306 in late S/G2-phase (green) remained green >90% of the time and did not progress into mitosis, indicating the action of RO-3306 is fast and arrests cells in late G2-phase. By 16 h, >90% of cells are green, consistent with G2-phase arrest via CDK1 inhibition (Supplementary Fig. S1d online)^18^,^19^.

### Cell cycle-associated death standards

**Figure 2:**
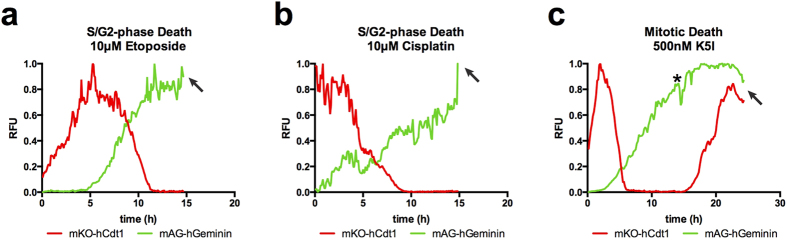
HT1080 FUCCI show strong cell cycle-associated cell death. Cell cycle-associated death standards are used. (**a**) A representative FUCCI trace of cells treated with the topoisomerase-α poison, etoposide. Cells progress from G1-phase (red), with normal kinetics, progress to a green state and die, consistent with S/G2-phase associated death. (**b**) A representative FUCCI trace of cells treated with the DNA modifier, cisplatin. Cells most often progress normally from G1-phase (red) to an all green state and die, consistent with S-phase associated death. (**c**) A representative FUCCI trace of cells treated with a Kinesin-5 inhibitor, K5I. This cell progresses through the cell cycle with normal kinetics and enters mitotic arrest at 14 h post-treatment (*). While arrested, red signal is reacquired after 3–4 h, beginning at 17 h. This cell dies at 23 h and nearly all other cells also die while arrested in mitosis. Arrows indicate time of death. See Supplementary Fig. 1e–g online for FUCCI distributions over time. Supplementary video S6-8 online. Cell number tracked: etoposide, 33, cisplatin, 21, K5I, 30.

Many anti-cancer drugs trigger cell cycle-associated killing in S-phase or mitosis^20^,^21^. Two clinically relevant agents with S-phase associated activities are the topoisomerase-IIα inhibitor, etoposide (VP-16)^22^,^23^, and the platinum-based chemotherapeutic, cisplatin^24^,^25^. Etoposide locks catalytically active topoisomerase IIα (TOP2A) onto the DNA, nicking both strands but preventing replication fork progression, manifesting as double-strand breaks. Cisplatin binds DNA and causes cross-links and subsequent double-strand breaks. Both etoposide and cisplatin strongly induce apoptosis. G1-phase cells treated with 10 μM etoposide progress normally into early S-phase, become green and remain green until their death (Fig. 2a, Supplementary video S6 online). By 24 h, ~90% of surviving cells are green (Supplementary Fig. 1e online). Cisplatin treated (10 μM) G1-phase cells progress into S-phase and show strong S-phase phenotypes and associated cell death (Fig. 2b, Supplementary video S7 online). By 16 h, ~90% of surviving cells are yellow or green, consistent with a strong S-phase arrest phenotype (Supplementary Fig. S1f online). For both etoposide and cisplatin, cells that were treated in S/G2-phase (green) most often arrested, indicating these cells rapidly respond to the drugs. Each of these standard but mechanistically distinct S-phase-associated cytotoxic agents result in cell death overwhelmingly while cells are yellow or green, and surviving cells that arrest are also yellow or green (Supplementary Fig. S1f online).

To characterize specific perturbations of each cell cycle stage before investigating selinexor, we also used a mitotic inhibitor. We selected the mitotic motor, Kinesin-5 inhibitor (K5I) EMD534085 because it should not perturb cell cycle progression until mitotic entry^1^,^26^. We find that cell cycle progression kinetics are normal in 500 nM K5I until mitotic entry, when cells arrest and >90% die while arrested (Fig. 2c, Supplementary video S8 online). These data demonstrate that, as predicted, K5I does not perturb cell cycle until progression into mitosis. After 3-4 h of mitotic arrest and before death, all cells re-acquire red fluorescence without losing green fluorescence (e.g. Fig. 2). As a result, at 8 h 70% of cells are green, but this distribution shifts to yellow over time as red fluorescence is reacquired before cells die (Supplementary Fig. S1g and Supplementary video S8 online). We also observe this phenomenon in Taxol-treated cells in mitotic arrest (Supplementary video S9 online). These data are consistent with data from another group also using Taxol (paclitaxel) and the microtubule depolymerizing drugs nocodazole and plinabulin (NPI-2358)^27^.

### Selinexor results in rapid and lasting inhibition of nuclear export

**Figure 3:**
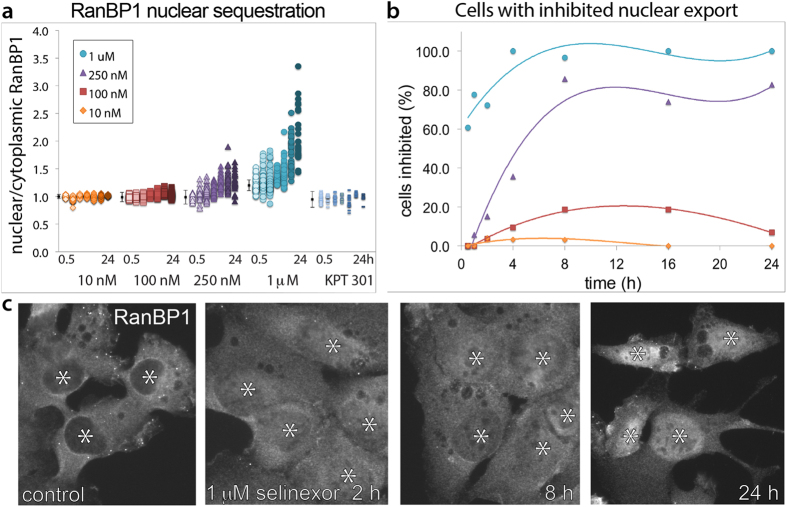
Selinexor causes rapid and persistent inhibition of nuclear export. (**a**) Scatter plot showing normalized (control) nuclear/cytoplasmic RanBP1 ratios in HT1080 cells for different selinexor concentrations. Cells were treated for: 0.5, 1, 2, 4, 8, 16 and 24 h. Error bars show the control average +/− two standard deviations for each experiment. Increased nuclear/cytoplasmic ratios are concentration and time-dependent. (**b**) Percent of HT1080 cells showing nuclear export inhibition after selinexor is concentration dependent. A cell is inhibited if its average nuclear/cytoplasmic RanBP1 ratio exceeds the average ratio in control cells by at least two standard deviations. Control uses an inactive SINE stereoisomer termed KPT 301. (**c**) RanBP1 staining in HT1080 cells for control and 2, 8, and 24 h. RanBP1 is rapidly sequestered and accumulates in the nucleus over time. Bar is 10 μm. Over 100 cells were measured for each condition.

Having established the specificity of FUCCI in our system, we sought to define the response to the nuclear export inhibitor, selinexor. Other studies show that selinexor results in sequestration of cargo proteins in the nucleus within hours (e.g. see refs 10,28,29). We confirmed selinexor action by immunofluorescent staining of RanBP1 and quantification of the nuclear to cytoplasmic ratio as a measure of nuclear sequestration (see Methods). RanBP1 is a key component of the XPO1 export machinery that shuttles between the nucleus and cytoplasm^30^,^31^,^32^. We treated cells with 10, 100, 250 and 1000 nM selinexor over time to establish dose and time dependence. Each condition was normalized to a non-treated control in parallel, and we generated a scatter plot of nuclear:cytoplasmic ratios (Fig. 3a). Based on significance tests (p < 0.05), we defined nuclear export to be inhibited when the mean nuclear:cytoplasmic ratio of RanBP1 exceeded that in control by two or more standard deviations, and we scored the inhibited cells over time (Fig. 3b). Treatment with 10 nM selinexor produced a small but measurable inhibitory effect at early time points that wanes rapidly after 8 h. There is a more significant effect at 100 nM selinexor, with peak inhibition of approximately 18% of cells at 8 h that waned significantly by 24 h. Inhibition is dose-dependent as at 250 nM we find that ~50% of cells are inhibited after 4 h and inhibition plateaus at ~80% of cells (Fig. 3b). We desired to inhibit nuclear export in all cells as rapidly as possible, so we increased the dose to 1 μM. After only 30 min, ~60% of cells show inhibition which increased to nearly 100% after 4 h and does not wane (Fig. 3b). Furthermore, nuclear:cytoplasmic ratios continue to increase over time and staining shows RanBP1 localization is overwhelmingly nuclear at 24 h (Fig. 3a,c). The SINE stereo-isomer KPT 301^9^,^28^ showed no sequestration of RanBP1 at 1 μM (Fig. 3a). To avoid potential effects due to a biological response, we generated HT1080 cells stably expressing an engineered XPO1 cargo, pmTurquoise2 fused to a canonical nuclear export sequence (NES)^33^. This arbitrary XPO1 cargo also underwent rapid nuclear sequestration upon 1 μM selinexor treatment (Supplementary Fig. S3 online). Based on our results, we defined 1 μM as our maximally effective dose. To minimize cell-to-cell variability because of incomplete or slow inhibition of nuclear export, and to ensure fast drug action allowing us to study cellular responses to acute selinexor treatment, we use 1 μM; other studies have also used 1 μM and there is no evidence of off target effects at this concentration^9^,^34^.

### Longitudinal tracking after selinexor reveals response heterogeneity and strong G1- and early S-phase phenotypes

**Figure 4:**
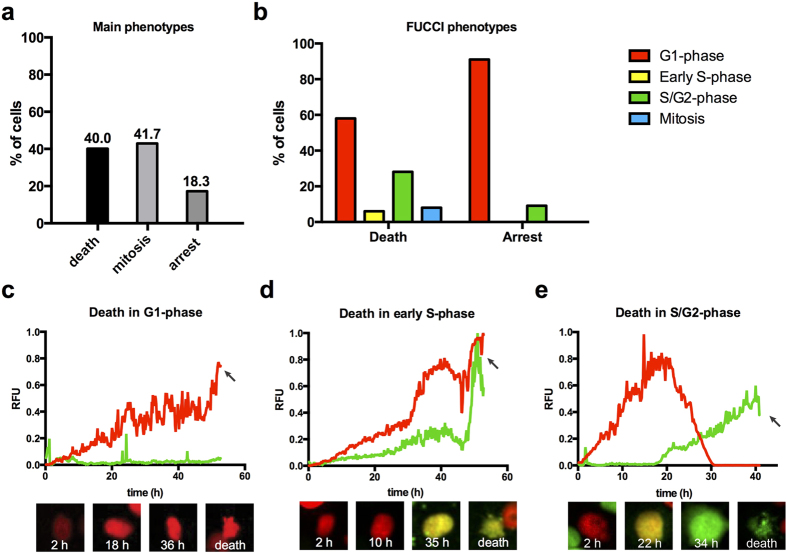
Selinexor results in strong, but highly variable cell cycle and cell death phenotypes. (**a**) The distribution of cell fates after selinexor treatment, as determined through longitudinal, single cell analysis. Approximately half of cells arrest or die, while the other half divides in the presence of drug. (**b**) The distribution of FUCCI populations at time of death and when arrested, respectively. 60% of death occurs in G1-phase and >90% of arrested cells are in G1-phase. Death in early S-phase and late S/G2-phase are also observed. (**c**–**e**) Representative examples of death from different cell cycle phases. (**c**) Death in G1-phase. This cell is tracked from birth, progressed into G1-phase and dies at ~52 h, indicating the cell is arrested in G1-phase prior to death. (**d**) Death in early S-phase. This cell is tracked from birth, progresses to yellow state after ~10 h, and remains yellow until the cell dies at ~52 h. (**e**) Death in late S/G2-phase. This cell is tracked from birth. After an abnormally long G1/early S-phase lasting ~30 h, it transitions to green and die ~41 h after birth. The inactive SINE stereoisomer KPT 301 has no effects. See Supplementary Fig. 1h, i online for FUCCI distributions over time. Arrows indicate time of death. Supplementary videos S11-13 online. (**a**,**b**) Cell number scored, 376. (**c**–**e**) Total cell number tracked, 117.

Non-synchronized FUCCI cells were treated when approximately 50–60% confluent with 1 μM selinexor or 1 μM KPT 301 and imaged every ten minutes for as long as 70 h. KPT 301 treated cells behaved like untreated with the same FUCCI profile (Supplementary Fig. S1h and Supplementary video S10 online). KPT 301 treated cells divided and grew to become confluent and background cell death was nil. Selinexor treated cells do not grow to become confluent and showed three phenotypes: death from interphase (37%), progression to and through mitosis (42%), and interphase arrest (18%), (Fig. 4a, Table 1). For those cells that died, nearly 58% died in G1-phase, 6% died in early S-phase (yellow), 28% died in S/G2-phase, and 8% died while in mitosis (Fig. 4, Table 1). For those cells that arrested in interphase, 91% were in G1-phase at the end of the time-lapse (Fig. 4b, Table 1). After 16 h of selinexor, ~80% of cells are in G1-phase (red), and the population remained in this state until 48 h, indicating little continued proliferation (Supplementary Fig. S1i online). Taken together, selinexor resulted in significant cell death and cell cycle arrest, particularly in G1/S-phase.

FUCCI markers have been multiplexed with cell death (apoptosis) markers and analyzed by flow cytometry^35^. To further establish the FUCCI status of dead cells, we performed a fixed time-point analysis of FUCCI cells stained with two indicators of apoptosis. Cytochrome C loss from mitochondria is an early event in apoptosis referred to as mitochondrial outer membrane permeabilization (MOMP) and caspase-dependent cleavage of poly-ADP ribose polymerase-1 (PARP1) is a later indicator of apoptosis. We find with both markers that the majority of scored dead cells were present in G1- and early S-phase, particularly at the 8 and 48 h time-points; the exact time of death in this assay is not known (Supplementary Fig. S4 online).

To effectively create a synchronized cell population, we used longitudinal tracking of the daughter cells resulting from the initial dividing population. Most of these divisions occur within the first 12 h of the time-lapse from cells treated in S/G2-phase. All cells born in selinexor progress to G1-phase. Of these, 64% subsequently die and 36% arrest in interphase (Table 1). Eighty-one percent of the death occurs in G1-phase after a protracted period of red fluorescence, compared to the approximately 4–6 h of G1-phase in untreated cells (Fig. 4c, Supplementary video S11 online). Two percent of the daughter cells die in early S-phase (yellow, Table 1) and this occurs after many hours of G1-phase (Fig. 4d, Supplementary video S12 online). Sixteen percent of cells that die progress through an abnormally long G1- and early S-phase lasting up to 30 h, become green, and then die within 8 h (Fig. 4e, Table 1, Supplementary video S13 online). In untreated and KPT 301 treated cells, the period of yellow indicating early S-phase lasts ~6–8 h; some selinexor treated cells remain yellow for over 20 h (Fig. 4d,e). Of the 36% of cells that arrested after dividing, 96% are in G1-phase (red) for the remainder of the time-lapse (Supplementary video S14 online) and 4% are late S/G2-phase green (Table 1).

We extended our analyses and tracked the progression and fates of cells treated in specific cell cycle phases based on FUCCI. In a normal, growing population of cells, 47% are typically in G1-phase, 13% are in early S-phase and 40% are in S/G2-phase (Supplementary Fig. S1a online). Of the cells treated in G1-phase, 53% die and 22% arrest (Supplementary videos S15-17 online), compared to 64% and 36% in the daughter cell population born in selinexor (Table 1). Twenty-five percent of cells treated in G1-phase (red) progress to mitosis and divide (Table 1). When early S-phase cells are tracked, we discover that like G1-treated cells, they are more sensitive to death than those treated in S/G2-phase (green, see below); 32% of them die (e.g. Supplementary video S18 online) and 4% arrest (Table 1). While 64% of treated, early S-phase cells divide (Table 1), most of the daughter cells die in the subsequent G1-phase. When S/G2-phase treated cells are followed, we find that 98% progress to mitosis and 2% die green—no cells arrest (e.g. Supplementary video S14 online). Of those that progress to mitosis, some remain in S/G2-phase for very long times (e.g. 20 h), and others for very short times (e.g. 2 h), indicating that some are in late G2-phase when treated and do not incur any significant arrest or progression defects prior to progressing to mitosis.

To directly test if selinexor treatment perturbs the progression of late G2-phase cells to mitosis, we synchronized cells with RO-3306 for 16 h, washed and released them into normal medium, 1 μM KPT 301, or 1 μM selinexor. After release, selinexor-treated G2-phase cells progressed like those released into normal or KPT 301 medium and 50% of cells entered mitosis within 1 h, and >90% of the synchronized population progressed through mitosis in either condition by 12 h (Supplementary Fig. S5 online). These data indicate selinexor does not prevent cells from progressing from late G2-phase into mitosis. Taken together, selinexor is a fast acting molecule that induces strong effects on G1- and early S-phase cells and little effect on G2-phase cells, and results in either long-lasting cell cycle arrest or death, most often in G1- and early S-phase.

### Population survival analyses using longitudinal tracking indicate G1- and early S-phase sensitization to selinexor

**Figure 5:**
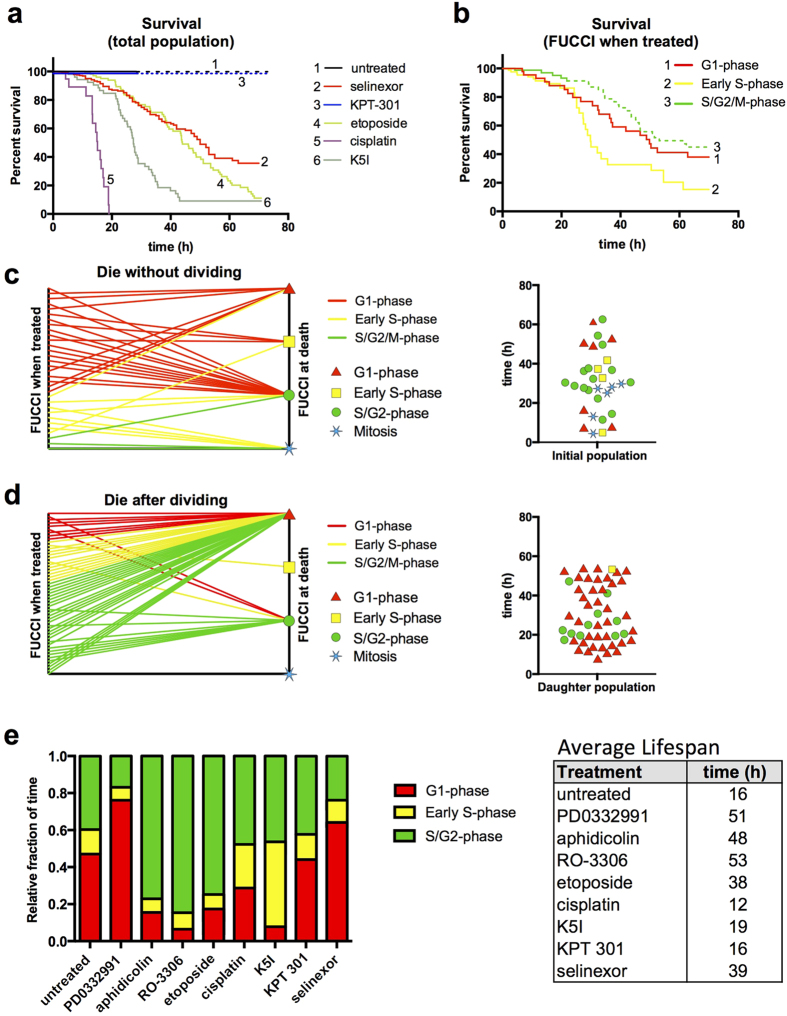
Longitudinal single cell tracking with survival analysis reveals cell cycle-associated responses of selinexor. HT1080 FUCCI cells. (**a**) Percent survival after treatment with cell cycle drugs, selinexor and controls. 100% of cells have divided by ~16 h for untreated (black) and KPT 301 treated (blueberry) cells; dashed lines represent the daughter cell population. Half of selinexor-treated cells are lost by ~55 h (maraschino) and the rate of loss is most similar to the S-phase associated drug, etoposide (honeydew); cisplatin (grape) and K5I (avocado) are comparatively very potent killers. (**b**) Survival curve for selinexor treated cells separated by FUCCI status upon treatment. Cells treated in early S-phase (yellow) die the fastest. Cells treated in late S/G2-phase (green) show little death and instead divide (dashed green line). Cells treated in G1-phase (red) and daughter cells from treated late S/G2-phase cells die at very similar rates. (**c**,**d**) Two-axis and violin plots for all cells that die after selinexor treatment or that die after being born into selinexor. Two-axis plots show FUCCI status upon treatment on the left axis and upon death on the right. Violin plot shows timing of death and FUCCI status (red triangle for G1-phase, yellow square for early S-phase, green circle for S/G2-phase, and blue star for mitosis) upon death. For (**d**) the FUCCI status of parent cells upon treatment are on the left axis and FUCCI status of daughter cells upon death on the right –84% of cells that die after dividing in selinexor, die in G1-phase (~84%). (**e**) Continuously tracked cells to obtain fraction of time spent in each FUCCI stage for each condition and table indicating the average life-span of cells for each condition; selinexor treated cells live 42 h on average, and spend increased time in G1-phase in particular (see Table 1). Supplementary videos S15-18 online. Cell numbers scored: (**a**–**d**) untreated, 42, selinexor, 376, KPT 301, 47, etoposide, 84, cisplatin, 54, K5I, 51. (**e**) Cell number tracked: untreated, 22, PD0332991, 19, aphidicolin, 24, RO-3306, 20, etoposide, 33, cisplatin, 21, K5I, 30, KPT 301, 27, selinexor, 117.

To distill these variable single cell responses into a population level response, we plotted cumulative survival curves^1^,^6^. Untreated and KPT 301 treated cells show no population loss and normal proliferation (Fig. 5a). Selinexor treatment results in loss of half of the entire population, and most of its cell death by approximately 50 h. For comparison, we plotted survival curves for the S-phase associated cytotoxic agents etoposide and cisplatin, and the mitotic arrest agent, K5I (Fig. 5a). Etoposide shows a loss of cells similar to selinexor, but with less survival. Cisplatin and K5I show rapid and greater cell loss.

To investigate the proliferative capacity of the selinexor surviving population, 24 and 48 h treated cells were plated thinly and followed using a colony formation assay that allows for cell scoring and FUCCI quantification (see Methods). Twenty-four and 48 h were chosen because time-lapse analyses indicate there are significant cell cycle effects by 24 h but with little population loss, and by 48 h cell cycle distribution of the surviving cells hasn’t changed significantly, but there is considerable cell loss (Fig. 5a, Supplementary Fig. S1i online). Neither condition recovered well, indicating that although selinexor is removed when the cells are alive, they have committed to arrest or death by 24 h, and if cells do divide, the progeny must not be viable (Supplementary Fig. S6a online). The starting FUCCI green:red ratio before selinexor release was consistent with other quantification (Supplementary Figs S6b and S1i online) and remained significantly lower than in control cells throughout the recovery period. We also performed a recovery assay without replating. This experiment starts with a higher cell number, and documents cell loss following removal of selinexor after 24 and 48 h treatment, and little recovery over one week in normal growth medium (Supplementary Fig. S6c, d online). To extend these findings, we measured selinexor-treated cells for the proliferation indicators, Ki67 and mitotic index, at 24 and 48 h. In strong agreement with our longitudinal FUCCI data and colony formation assay, Ki67 and mitotic index drop precipitously after selinexor, and are dramatically decreased by 48 h indicating a non-proliferative state (Supplementary Fig. S7 online).

Next, we parsed out sub-populations of cells that were treated in G1-phase, early S-phase, or S/G2-phase and plotted cell survival kinetics. Death appears to occur earlier and faster for cells treated in G1- and early S-phase compared to those treated in S/G2-phase, with early S-phase being particularly sensitive (Fig. 5b, lines 1 and 2). For cells treated in early S-phase, 50% are lost by 30 h, compared to 50 h for cells treated in G1-phase. Due to a higher percentage of early S-phase treated cells that divide after being treated, compared to G1-phase (64% vs. 25%, Table 1), we see a much steeper decline in survival between 25 and 35 h post-treatment because many of the daughter cells are dying in the next G1-phase (Fig. 5b, line 2). Death of cells treated in S/G2-phase is initially dramatically less because many of these cells divide within the first several hours (Fig. 5b, line 3). However, the daughter cells die in the subsequent G1-phase at a rate very similar to the loss of the cells directly treated in G1-phase (Fig. 5b, dashed green line 3).

G1- and early S-phase treated cells are more likely to die than those treated in S/G2-phase (Fig. 5b, Table 1). To test for relationships between cell cycle phase when treated and cell cycle phase upon death, we plotted which phase cells were in when treated and which phase they were in when they died on a two axis plot, and the phase and time of death in a violin plot for cells that died without first dividing (Fig. 5c). While some cells die in the same stage as treated, there is variability in both the cell cycle stage and timing of death and no obvious relationship. The same analysis that tracks daughter cells born in the presence of selinexor, reveals that >80% of those that die, do so in the next G1-phase (Fig. 5d, Table 1). This indicates that if a cell goes through mitosis in the presence of selinexor, there is a strong likelihood that it will die in the subsequent G1-phase. Fixed cell scoring in Supplemental Fig. 4 online agrees well with time-lapse data, that more death events from G1-phase occur early and late after selinexor treatment.

The impact of different treatment conditions on the relative fraction of time spent in each FUCCI phase and on the lifespan of cells was determined (Fig. 5e). Selinexor treated cells survive on average 39 h compared to a 16 h cell cycle in untreated cells, and spend increased time especially in G1-phase compared to all other conditions except PD0332991 treated cells (Fig. 5e).

The ability of HT1080 cells to arrest in each cell cycle phase is strong, and therefore the variability of the single cell phenotypes after selinexor isn’t due to a lack of ability to exert a checkpoint per se, but is instead likely due to complex checkpoint and stress pathways in response to selinexor that may be sensitized in G1- and early S-phase. Data that support this are that cells treated in G1- and early S-phase generally responded strongly with cell cycle arrest and death (Fig. 5, Table 1), but cells treated in G2-phase progress apparently without delay into mitosis (Fig. 5b, Supplementary Fig. S5 online), and don’t die or arrest until in the subsequent G1- and early S-phase. Together with reporters like FUCCI, single cell longitudinal tracking provides a powerful approach to study anti-cancer drug response.

## Discussion

How individual cancer cells respond to therapy can profoundly influence the overall outcome, either directly, for example through strong cytotoxic and cytostatic effects, or indirectly by impacting the tumor environment in a manner that is either pro-or anti-tumorigenic^36^,^37^,^38^. It is also important to understand single cell responses and precise relationships between cell response and cell fate as this information speaks to drug mechanism and can extend toward developing drug combinations that enhance therapeutic impact, or inform on combinations that will not be beneficial. Despite its importance, the types and distribution of single cell responses are often unknown—we used longitudinal, single cell tracking to study cell cycle effects of selinexor, a novel anti-cancer agent currently in multiple clinical trials^7^.

FUCCI are dynamic sensors that report on cell cycle stage and cell cycle progression. The CDK4/6 inhibitor PD0332991 does not prevent S/G2-phase cells from progressing through mitosis, as treated green cells first divide and then arrest red (e.g. Fig. 1d). We observe approximately 10% of red cells transition to a green state and then revert back (Supplementary video S3 online). This observation has now been made in HT1080 and mouse NMuMG cells^13^, and in endoreduplicating cells during normal development in FUCCI transgenic mice^39^, that are each p53 wildtype; reversion did not occur in HeLa^40^, that are dysfunctional for p53.

Kinesin-5 inhibitor does not perturb cell cycle progression until cells enter mitosis (Fig. 2c). After mitotic arrest, cells are trapped, remain green, and >90% die while arrested (Fig. 2c, Supplementary video S8 online). During mitotic arrest, cells acquire increasing red fluorescence (Fig. 2c, Supplementary video S8 online). This is also observed with the microtubule stabilizer Taxol (Supplementary video S9 online and ref. 27), and microtubule depolymerizers nocodazole, KPU133 and plinabulin^27^,^40^. Because this acquisition of red fluorescence occurs for mechanistically distinct drugs, and is observed by multiple groups using different cell lines, we contend it is not a drug-induced anomaly, but rather indicates a decrease in SCF2 E3-ubiquitin ligase activity, allowing for the accumulation of the red probe. We note here, the timing of the red fluorescence during mitotic arrest is similar with K5I and Taxol in HT1080, and with plinabulin, KPU133, nocodazole, and Taxol in HeLa (3–6 h)^27^. This suggests there is a background clock in mitotic arrested cells, where they begin to transition to a G1-like state when held there long enough. Ours and data from Honda-Uezono *et al.* and Kaida *et al.* are consistent the ‘competing pathways’ hypothesis during mitotic arrest^2^,^41^, and provide a refinement: during prolonged mitotic arrest, the activity of the spindle assembly checkpoint and apoptosis pathways progress in parallel, and the decision to slip to a post-mitotic state or die from arrest is dependent on when a commitment threshold is reached, and cells begin to transition toward a post-mitotic G1-state during protracted arrest, prior to slippage.

Selinexor action is unique compared to many anti-cancer agents, including the etoposide, cisplatin, and K5I used here. Many of these agents work by perturbing enzyme function, for example etoposide converts TOP2A into a DNA damaging endonuclease, and K5I blocks the mitosis specific function of Kinesin-5. Cisplatin directly modifies the DNA, resulting in damage. Selinexor inhibits a single protein’s function, resulting in the nuclear sequestration of many cargo proteins^34^. Selinexor blocks the nuclear export of p53, pRB, p21^Cip1^, and p27^Kip1^ and other proteins including, TOP2A, PCNA, and survivin, that can each impinge on cell growth and cell death regulatory pathways^8^,^42^,^43^. Exportin-1 is expressed in all cell cycle stages^44^, making it an attractive target for both fast and slow growing cancers. Most cells show inhibited nuclear export by 2 h at 1 μM (Fig. 3). Thus, cell cycle-specific responses can be studied after acute selinexor treatment. The data indicate that selinexor action is strongly associated with G1- and early S-phase. S/G2-phase cells treated with selinexor progress through mitosis first, and the daughter cells respond in the subsequent cell cycle, usually in G1-phase (Fig. 5, Table 1, Supplementary videos S11-14 online).

The S/G2-phase cells that divide after selinexor do not represent a drug resistant population, as they do show strong nuclear export inhibition based on RanBP1 accumulation in nearly all cells by 4 h. Rather, it indicates these cells cannot commit to cell cycle arrest or death the same as cells in G1- and early S-phase and/or that selinexor gains efficacy via a process or stress response that occurs in G1- or early S-phase, and not in late S- or G2-phase.

*In situ,* many factors influence therapeutic response, including overexpression of the drug target, drug delivery, proliferation rate, and tumor microenvironment. Exportin-1 is overexpressed in different cancers, including ovarian^45^ and pancreatic^46^, and correlates with poor survival and decreased response to standard of care therapy, but if and how XPO1 overexpression affects selinexor response is currently unclear. Our data suggest selinexor may exert anti-cancer effects on slow growing tumors, where the bulk of the cell mass is represented by a G1-like state^36^,^47^. Our data in HT1080, and others’ studies, indicate strong responses to selinexor occur in cancer-derived cells with an intact p53-dependent G1/S-phase checkpoint, perhaps suggesting that cells and cancers with and intact G1/S checkpoint may predict strong response^11^,^43^,^48^,^49^. However, in the absence of p53, cancer cells and xenograft tumors can respond, indicating that p53 may be dispensable for response^9^,^11^,^43^. Given the nature of selinexor action to effectively sequester many different proteins within the nucleus, and that XPO1 has multiple functions, it is possible XPO1 inhibition perturbs cells in multiple ways, resulting in cytostatic and cytotoxic effects. For example, without a potent G1/S-phase checkpoint, cells may die via accumulated genotoxic stress due to proliferation with a lack of robust arrest and/or diminished DNA damage repair, which is evident in many tumor lines. Another contributing factor to anti-cancer response is death sensitivity, which varies widely across cancer-derived cell lines^50^,^51^. Further, within a given cancer, and between cancers, there is tremendous variability in the fraction of cells that proliferate, cell doubling-times, and the time spent in each cell cycle phase and these are often not known in patients. This variability may necessitate complex treatment schedules that would ideally be personalized to the cancer. Our data showing lack of proliferative capacity (Supplementary Figs S5 and S6 online) and other data showing decreased Ki67, and high nuclear p27^Kip1^ and p21^Cip1^ in residual xenograft tumors, indicate that some cells may enter a senescent state after selinexor^10^,^11^,^52^,^53^. In the tumor microenvironment, surviving, senescent tumor cells can exert pro- and anti-cancer mechanisms^54^,^55^. In future studies it is important to test the effect of selinexor on slow growing tumors, the roles of specific XPO1 cargos, and to study the impact of the selinexor-induced senescent cell population on long-term tumor-level response and re-lapse.

## Materials and Methods

### Cell line growth and genesis

Cell lines are maintained at 37 °C, 5% CO_2_, 80% humidity. HT1080 and HEK293FT cell lines are grown in MEM with EBSS, and DMEM high glucose medium (ThermoScientific) respectively. All media are supplemented with 10% v/v FBS (Sigma) and 1% v/v Pen/Strep (Sigma). MEM is also supplemented with 1% v/v sodium pyruvate (ThermoScientific), and 1% v/v non-essential amino acids (ThermoScientific). FUCCI expressing clones are stably integrated via lentiviral transduction. FUCCI encoding lentiviral plasmids are from Sakaue-Sawano *et al.*^4^, via material transfer agreement; mKO2-hCdt1(30/120) [DDBJ/EMBL/GenBank, AB370332] in pCSII-EF vector and mAG-hGem(1/110) [DDBJ/EMBL/GenBank, AB370333] in pCSII-EF, respectively. FuGene6 (Promega) was used to transfect pmTurquoise2-NES into cells.

### Small molecules

KPT 330 (selinexor) and inactive KPT 301 are from Karyopharm Therapeutics, Inc. (Natick, MA) and dissolved in anhydrous cell culture-grade dimethyl sulfoxide (DMSO, Sigma). PD0332991, Etoposide (VP-16), and cisplatin are from Selleckchem (Houston, TX); stock solutions re in DMSO except for cisplatin, which is in dimethylformamide. Aphidicolin (Sigma) is dissolved in DMSO. RO-3306 (Enzo Life Sciences, San Diego, CA) is dissolved in DMSO. The K5I EMD534085 is from Merck Serono (Darmstadt, Germany) and dissolved in DMSO. All small molecules are diluted into pre-warmed growth media to final desired concentration just prior to use.

### Immunofluorescent staining

Cells are grown to 70% confluence on 70% ethanol washed and autoclaved, #1.5 coverslips (VWR). Cells are fixed in 3.7% paraformaldehyde (Mallincrokdt) in PBS for 20 min at room temperature and permeabilized with 0.2% Triton X-100 (Sigma) in PBS for 5 min at room temperature. Cells are blocked in 4% BSA fraction V ((ThermoScientific) in PBS for 1 h at room temperature before immunofluorescent staining. RanBP1 is detected with anti-RanBP1 (polyclonal goat, Santa Cruz Biotechnology, sc-1160) at 1.0 μg/ml in 4% BSA/PBS for 1 h at room temperature. Ki67 is detected with anti-Ki67 (rabbit polyclonal, Santa Cruz Biotechnology, H-300 sc-15402) at 4 μg/ml in 4% BSA/PBS for 1 h at room temperature. Coverslips are washed 5 times in PBS and anti-goat or anti-rabbit Alexa488 (LifeTech) secondary antibodies are used at 2 μg/ml in 4% BSA/PBS for 1 h at room temperature. Coverslips are counter-stained with 10 μg/ml Hoechst 33342 (bis-benzimide, Sigma) or DAPI (LifeTech) in PBS for 5 min at room temperature. Coverslips re washed 5 times in PBS, 2 times in distilled water, and mounted onto Prolong Gold anti-fade using 70% ethanol-cleaned and dried glass Gold Seal or VWR microscope slides, and cured at room temperature overnight in the dark.

### Microscopy

Cells are grown to approximately 50–60% confluent on 35 mm glass bottom dishes (MatTek or *InVitro* Scientific) before imaging. Live-cell time-lapse imaging is performed using an automated Olympus IX81 inverted widefield microscope equipped with a motorized stage (Prior Proscan III, Prior Scientific), Hamamatsu ORCA-R2 CCD camera, 200 watt LumenPro200 metal halide lamp (Prior Scientific) and customized stage-top incubation system (*InVivo* Scientific) at 37 °C and 90% humidity using Slidebook6 (3I). Olympus objectives used are 10 X 0.40PH2, 20X/0.7NA PH2 UPLAPO Plan Apo and 40X/0.75NA PH2 UPLFLN SemiApo. Time-lapse microscopy images are taken every 10 min using mCherry and EGFP excitation/emission and phase-contrast microscopy. Autofocusing is applied in the transmitted light channel, images are binned 2 × 2 and fluorescent exposures are 10–30 ms. Spinning disc confocal time-lapse microscopy is from an Olympus CV1000 equipped with a spinning disc confocal head and environmental control at 37 °C and 80% humidity; images are every 10 min using 488 nm and 568 nm laser illumination, a red LED for transmitted light, and an Olympus 20 X 0.75NA objective. Fixed-cell images are acquired using mCherry, EGFP, Cy5/Aleca647 and/or Hoechst/DAPI illumination; imaging conditions are identical between all imaging sessions.

### Cell recovery assays

For the colony formation assay, HT1080 FUCCI cells treated with 0.5% DMSO (control) or 1 μM selinexor for 24 and 48 h, are washed 5 times in pre-warmed medium, trypsinized, counted and 10,000 are plated into normal growth medium in 35 mm gridded MatTek dishes. Twelve marked positions in each grid that contained a cell are imaged during recovery with a 10X objective and phase-contrast and fluorescence; the same positions are followed throughout the recovery. The number of cells at each position are scored by overlaying the phase-contrast and fluorescence images and counting nuclei. Red and green FUCCI signals are measured per position and green:red ratios determined using Excel (Microsoft). Relative recovery is determined by normalizing the cell number in each position to the average cell number of the 12 control positions when they became confluent. For the cell recovery assay, 30,000 cells were plated into gridded MatTek dishes and grown for two days prior to selinexor addition. Twelve locations were randomly chosen and imaged before selinexor, after selinexor, and every 24 h during recovery. Analysis was the same as the colony formation assay. Plots of relative recovery and FUCCI ratios are produced in Excel.

### Cell tracking, FUCCI, RanBP1 and Ki67 quantitative analysis

Individual FUCCI cells are identified and regions of interest (ROI) are defined within the nucleus of each cell and cells are tracked visually in every frame for their duration. ROIs are saved for each position and time point. Cell phenotypes and fates and the timing of the response are scored for each cell and logged into an Excel spread sheet. Mitotic entry is marked by observing nuclear envelope dissolution concomitant with chromatin condensation and cell rounding. Apoptosis is marked by morphological hallmarks including blebbing, rounding-up, and cessation of normal cell dynamics. The mean FUCCI red and green fluorescent intensity within each ROI at every frame is quantified using ImageJ (NIH) software, intensities were normalized to the average range observed in untreated cells and FUCCI status was calculated using Excel, and plots are generated using GraphPad Prism software. FUCCI signals in each cell are normalized to their peak intensity to represent a percentage of the average range of fluorescence in untreated cells.

To calculate nuclear:cytoplasmic RanBP1 ratios, nuclei ROIs are identified using thresholding in the DAPI channel and the analyze particles function of ImageJ. The mean, nuclear intensity of the RanBP1 signal is calculated using the measure function of ImageJ; cytoplasmic intensity was calculated as the mean of 10 cytoplasmic sample points per cell. Nuclear export inhibition is defined as a nuclear:cytoplasmic ratio exceeding the mean value from control cells by at least two standard deviations (p < 0.05). Plots of nuclear:cytoplasmic ratios and percent inhibited cells are generated using Excel. For Ki67 scoring, nuclei are identified using the DAPI stain and ROIs confined in each nucleus are defined and the mean fluorescence intensity in each ROI is measured. Background fluorescence is subtracted and cells are scored as positive or negative for Ki67 following a published method^56^.

## Additional Information

**How to cite this article**: Marcus, J. M. *et al.* Longitudinal tracking of single live cancer cells to understand cell cycle effects of the nuclear export inhibitor, selinexor. *Sci. Rep.* **5**, 14391; doi: 10.1038/srep14391 (2015).

## Supplementary Material

Supplementary video S1

Supplementary video S2

Supplementary video S3

Supplementary video S4

Supplementary video S5

Supplementary video S6

Supplementary video S7

Supplementary video S8

Supplementary video S9

Supplementary video S10

Supplementary video S11

Supplementary video S12

Supplementary video S13

Supplementary video S14

Supplementary video S15

Supplementary video S16

Supplementary video S17

Supplementary video S18

Supplementary Information

## Figures and Tables

**Table 1:** Cell fates and FUCCI association after selinexor.

	Treatment	
	Untreated	selinexor
Cell fate determination (all cells)	% of total population	
Death	0%	40%
Mitosis	100%	42%
Arrest	0%	18%
FUCCI status - cells that die (all cells)	% of death population	
G1-phase (hCdt1 only)	—	58%
early S-phase (hCdt1 and hGem)	—	6%
mid-late S/G2-phase (hGem only)	—	28%
mitosis (hGem only)	—	8%
FUCCI status - cells that arrest (all cells)	% of arrested poplulation	
G1-phase (hCdt1 only)	—	91%
early S-phase (hCdt1 and hGem)	—	0%
mid-late S/G2-phase (hGem only)	—	9%
Cell fate determination (daughter cells)	% of G1-phase cells	
Death	0%	64%
Mitosis	100%	0%
Arrest	0%	36%
FUCCI status - cells that die (daughter cells)	% of death population	
G1-phase (hCdt1 only)	—	81%
early S-phase (hCdt1 and hGem)	—	2%
mid-late S/G2-phase (hGem only)	—	16%
mitosis (hGem only)	—	0%
FUCCI status - cells that arrest (daughter cells)	% of arrested poplulation	
G1-phase (hCdt1 only)	—	96%
early S-phase (hCdt1 and hGem)	—	0%
mid-late S/G2-phase (hGem only)	—	4%
Fate determination - cells treated in G1-phase	% of daughter cells	
Death	0%	53%
Mitosis	100%	25%
Arrest	0%	22%
Fate determination - cells treated in early S-phase	% of early S-phase cells	
Death	0%	32%
Mitosis	100%	64%
Arrest	0%	4%
Fate determination - cells treated in S/G2/M-phase	% of S/G2/M-phase cells	
Death	0%	2%
Mitosis	100%	98%
Arrest	0%	0%
Average time observed per cell cycle phase	time (hours)	
G1-phase (hCdt1 only)	5	23
early S-phase (hCdt1 and hGem)	2	5
mid-late S/G2-phase (hGem only)	7	18
Mitosis	1	2

The distribution of phenotypes in response to selinexor and the FUCCI-association with specific cell fates for different quantified cell populations are shown. Average time spent in each FUCCI stage is also provided. See Figures for cell numbers analyzed.
