# Supplementary material for: Longitudinal tracking of single live cancer cells to understand cell cycle effects of the nuclear export inhibitor, selinexor
Source: Sci Rep. 2015 Sep 24;5:14391. doi: 10.1038/srep14391 (PMC4585873; doi:10.1038/srep14391)
Supplement: Supplementary Information [file srep14391-s19.pdf]

Longitudinal tracking of single live cancer cells to understand cell cycle effects of the nuclear export inhibitor, selinexor

Joshua M. Marcus[1], Russell T. Burke[1], John A. DeSisto[1], Yosef Landesman[2], and James D. Orth[1*]

## Supplementary Figure S1

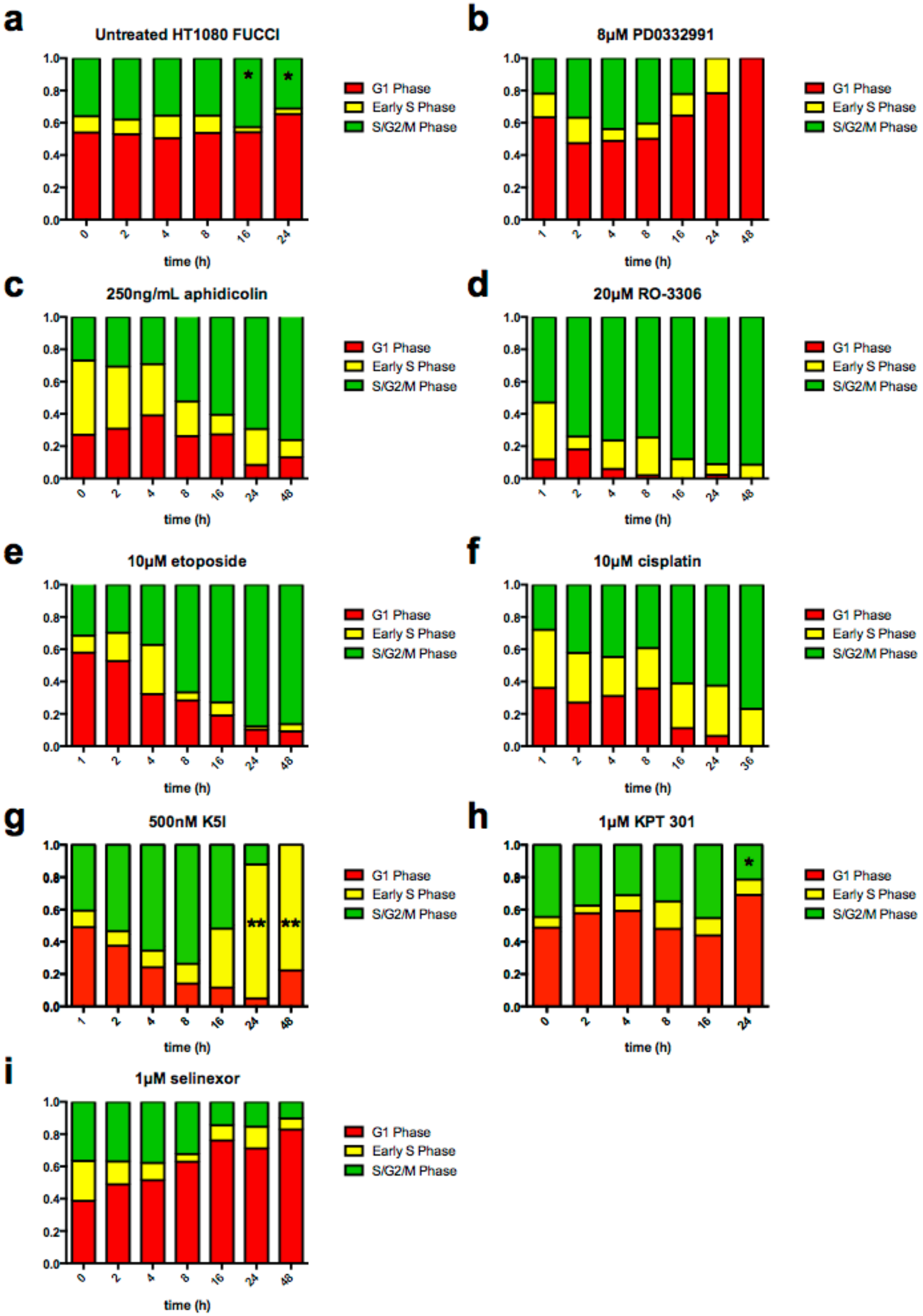

**Supplementary Figure S1. Fraction of FUCCI status measured over time for each condition.** Cells were treated at 50-60% density for all conditions. a) Untreated control cells become confluent by 16 h, resulting in a change in FUCCI distribution (asterisk). b) 8µM PD0332991. Some cells initially treated red, cycle through and divide and the daughter cells arrest, which is evident by the initial loss in red population between 1 and 2 h post treatment. c) 250ng/mL aphidicolin results in strong early (yellow) and late (green) S-phase arrest and 90% yellow or green cells at 48 h. d) 20µM RO-3306 results in strong G2-phase arrest and 90% green cells at 48 h. e) 10µM etoposide results in strong late (green) S-phase arrest and >80% of surviving cells are green cells at 48 h. f) 10µM cisplatin results in strong early (yellow) and late (green) S-phase arrest and nearly all surviving cells are yellow or green cells at 36 h. g) 500nM K5I does not block progression through interphase, cells accumulate prolonged mitotic arrest that is initially green by 4-8 h, and regain some red fluorescence during arrest, resulting in a large yellow population as cells die from mitotic arrest (double asterisk). At 48 h the few surviving interphase cells are yellow or red. h) 1µM of the inactive SINE KPT 301 has no effects; cells become confluent at 24 h (asterisk). i) 1µM selinexor results in complex responses (see text), and cells are 80% red by 16 h and the distribution remains that way until 48 h indicating little continued proliferation. Cells continue to be lost (Fig. 6a, b) and 80% of survivors that are non-proliferative (Supplementary Fig. 5, 6 online) at 48 h are red. Over 50 cells scored for each condition.

**Supplementary Figure S2**

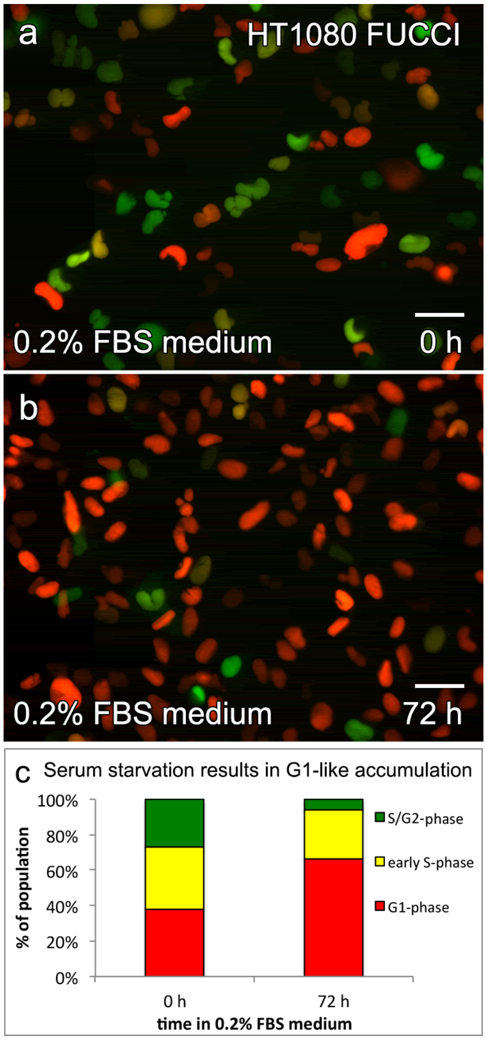


**Supplementary Figure S2. FUCCI cells shift to a less proliferative, red distribution in low serum conditions.** a) Immediately after cells are placed in 0.2% FBS medium, they show a high proliferative state with approximately 40% red, 30% yellow and 30% green nuclei. b) After 72 h of being maintained in 0.2% FBS medium, the population is mainly red, indicating a lack of proliferation with approximately 70% red, 25% yellow and only 5% green. Bars = 10μm. Over 100 cells scored for each condition.

**Supplementary Figure S3**

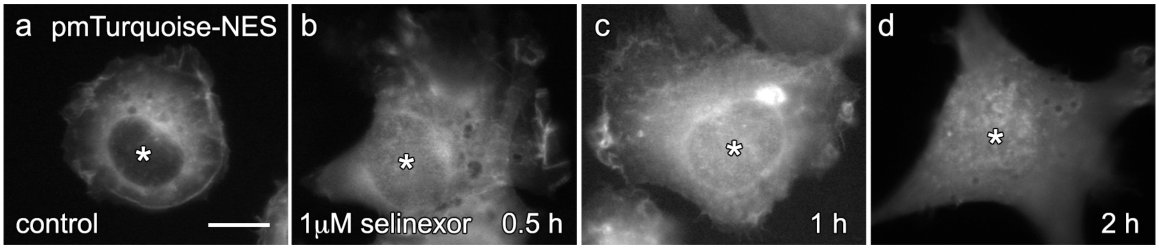


**Supplementary Figure S3. Selinexor causes rapid inhibition of nuclear export and nuclear sequestration of pmturquoise2-NES.** a) Before selinexor treatment, control cells show a predominantly cytoplasmic localization of the fluorescent fusion protein. b-d) Like for RanBP1, as soon as 0.5 h after 1μM selinexor and thereafter, the cargo protein becomes sequestered in the nucleus indicating inhibition of nuclear export. * mark nuclei. Bar = 10μm.

## Supplementary Figure S4

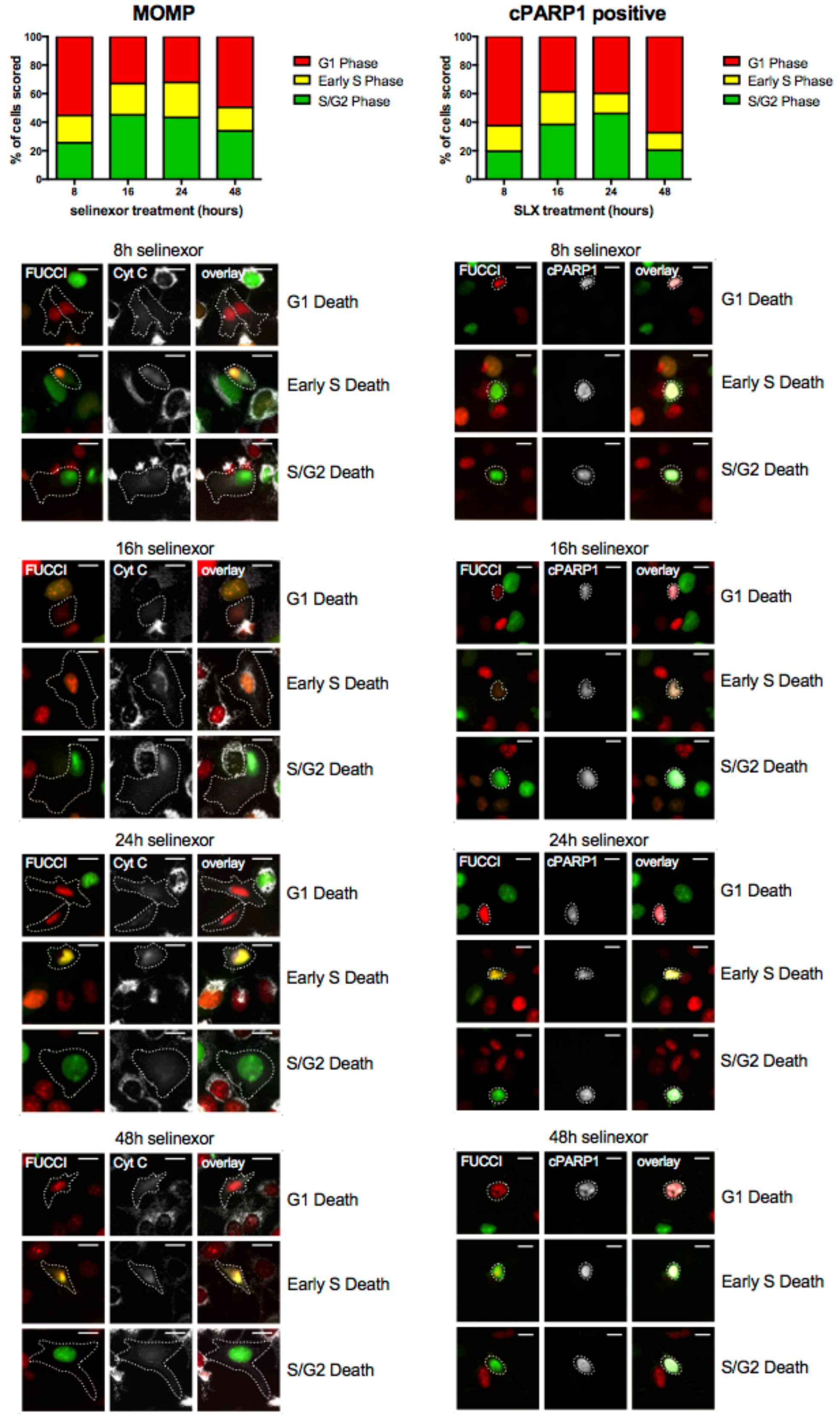

**Supplementary Figure S4. HT1080 FUCCI cells treated with 1μM selinexor for 8, 16, 24 and 48 h show more association with G1- and early S-phase apoptosis.** a) Treated cells were stained with a cytochrome C antibody to reveal cells that had undergone MOMP. At all time-points, cells showing MOMP are predominantly G1- and early S-phase. At the 8 and 48 h time-points over 50% of MOMP cells indicate G1-phase. Representative images of MOMP cells in G1-, early S-, and S/G2-phase are shown for each time-point. b) Treated cells were stained with a cleaved PARP1 antibody (cPARP1) to reveal cells that had undergone apoptosis. Like for MOMP cells, at all time-points, cells showing cPARP1 are predominantly G1- and early S-phase. At the 8 and 48 h time-points over 50% of cPARP1 cells indicate G1-phase. Representative images of cPARP1 cells in G1-, early S-, and S/G2-phase are shown for each time-point. DNA staining with DAPI and phase-contrast microscopy were also imaged to confirm the apoptosis phenotype. Over 80 cells were scored for each condition.

**Supplementary Figure S5.**

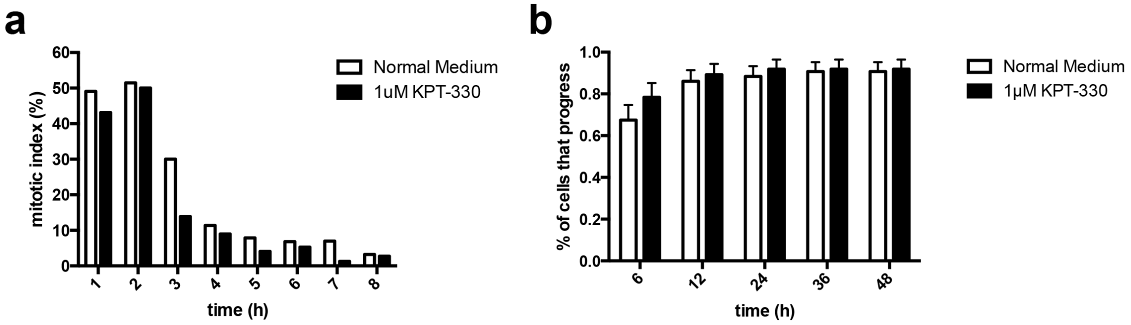


**Supplementary Figure S5. HT1080 FUCCI cells in G2-phase do not arrest upon inhibition of nuclear export**. Cells were synchronized in G2-phase, and released into normal medium or medium containing selinexor. a) Mitotic index was scored over time from 1 to 8 h after release. There was no evident effect of selinexor on release from G2-phase synchrony into mitosis. b) All cells were tracked longitudinally and scored for progression through mitosis after release into normal medium or 1μM selinexor. 70-80% of cells divide by 6 h release, and essentially all trackable cells in both conditions divide by 12 h. Over 60 cells tracked for each condition.

**Supplementary Figure S6**

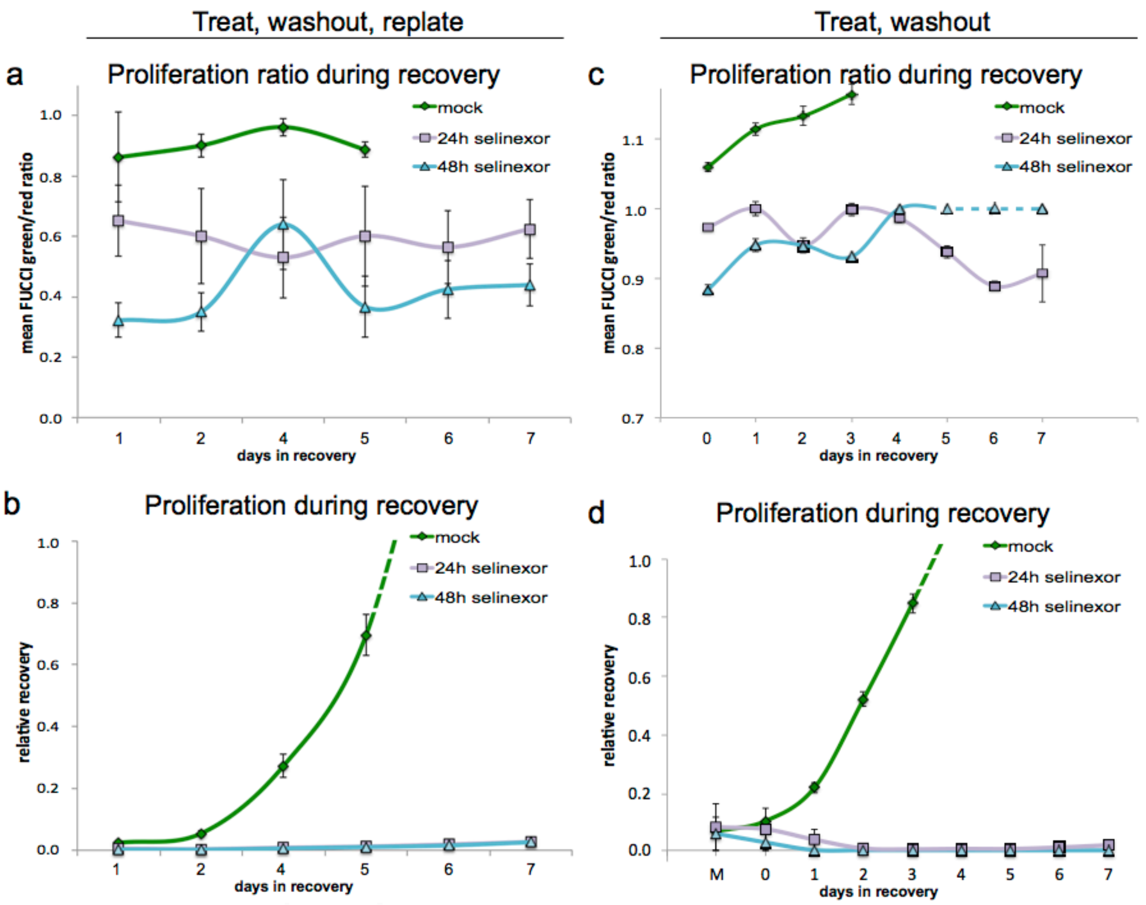


**Supplementary Figure S6. Recovery of HT1080 FUCCI cells after 24 or 48 h of selinexor treatment is poor.** a) Ten-thousand cells for each condition were re-plated into gridded glass-bottom dishes and quantified daily during recovery. Untreated control cells grew as colonies and showed a FUCCI green/red ratio of approximately 0.9 – 1.0 and they completely filled the dish by 5 days. 24 and 48 h show decreased green/red ratio indicating cell cycle effects of nuclear export inhibition that remained lower through 7 days. b) Recovery is determined by normalization to control cell number when confluent. Control cells are confluent by 5 days (dashed line), where cells treated with selinexor for 24 and 48 h are severely affected and show very little proliferative recovery, in

agreement with the constant and comparatively low green/red ratios (a). c) Thirty thousand cells were plated into gridded glass-bottom dishes and grown for two days before treatment with 1 μM selinexor. Cells were then treated for 24 or 48 h or left untreated. After treatment, cells were imaged and they were imaged and quantified daily during recovery. Untreated control cells grew immediately and showed a green/red ratio of 1.0 – 1.1 and they completely filled the dish by 3 days. After 24 and 48 h treatment, the green/red ration was decreased, especially after 48 h, indicating growth arrest as observed by time-lapse microscopy. For 24 and 48 h, the green/red ration remained lower through 7 days. After 5 days there were no cells remaining in the 48 h treated sample (dashed line). d) Control cells are confluent by 3 days (dashed line), where cells treated with selinexor for 24 and 48 h are severely affected and show very little proliferative recovery, in agreement with the constant and comparatively low green/red ratios (c). We note that in this assay we observe cell loss by 48 h treatment before wash-out, and the loss continues after selinexor wash-out.

**Supplementary Figure S7**

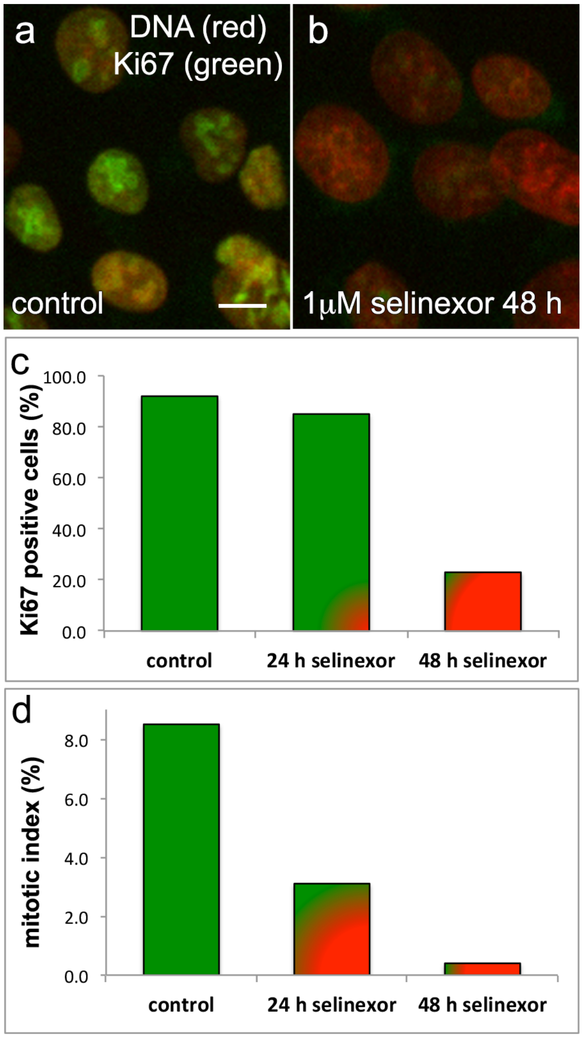


**Supplementary Figure S7. HT1080 cells lack proliferation markers upon 48 h of inhibited nuclear export.** a, c, d) Untreated control cells show high Ki67 staining in over 90% of cells, and over 8% mitotic index. c, d) 24 h treated cells show high remaining Ki67 likely due to protein half-life, but a reduced mitotic index of 3%. b-d) By 48 h treatment, Ki67 cells are dramatically reduced and the mitotic index is significantly less than 0.5%, indicating low proliferative capacity of cells in this condition. Bar = 10μm. Over 100 cells scored for each condition.

**Supplementary video legends:**

All videos captured with 20X 0.7 or 0.75NA objective. Images acquired every 10 minutes except videos S4, 7, and 9 which were acquired every 5 minutes. Representative cell is in center of each field.

Supplementary video S1.

HT1080 FUCCI cell tracked from early mitosis (green) through the cell cycle to a second round of mitosis. This cell shows transition through each cell cycle phase.

Supplementary video S2.

HT1080 FUCCI cell treated with 8μM PD0332991. Cell was treated in late G2-phase or early mitosis (green), divides normally and remains in G1-phase (red) for the balance of the video, indicating arrest.

Supplementary video S3.

HT1080 FUCCI cell treated with 8μM PD0332991. Cell was treated in G1-phase (red), progresses through the G1/S-phase checkpoint to late S/G2-phase (green), then transitions back to a red state after about 24 h and is arrested red for the balance of the movie.

Supplementary video S4.

HT1080 FUCCI cell treated with 250ng/ml of the DNA polymerase δ and ε inhibitor, aphidicolin. Cell was treated in G1-phase and progresses to S/G2-phase (green) for the balance of the movie indicating arrest.

Supplementary video S5.

HT1080 FUCCI cell treated with 20μM of the CDK1 inhibitor, RO-3306. Cell was treated in G1-phase and progresses to S/G2-phase (green) for the balance of the movie indicating arrest.

Supplementary video S6.

HT1080 FUCCI cell treated with 10μM etoposide. Cell was treated in G1-phase, transitions to early S-phase (yellow), then S/G2-phase (green), and dies while green.

Supplementary video S7.

HT1080 FUCCI cell treated with 10μM cisplatin. Cell is treated in G1-phase, transitions to early S-phase (yellow), then S/G2-phase (green), and dies while green. Phase-contrast unavailable.

Supplementary video S8.

HT1080 FUCCI cell treated with 500nM K5I. Cell is treated in G1-phase, progresses through to mitosis with normal kinetics, arrests in mitosis, becomes yellow after 3 h of arrest due to increased red fluorescence, remains arrested in mitosis and eventually dies.

Supplementary video S9.

HT1080 FUCCI cell treated with 100nM Taxol. A S/G2-phase cell 19 h after Taxol treatment progresses through to mitosis, arrests in mitosis, becomes yellow after 2 h 30 min of arrest due to increased red fluorescence, remains arrested in mitosis and eventually dies.

Supplementary video S10.

HT1080 FUCCI cell treated with 1μM KPT 301 tracked from very early mitosis, through to a second round of mitosis. This cell shows transition through each cell cycle phase.

Supplementary video S11.

HT1080 FUCCI cell treated with 1μM selinexor that is born into G1-phase. Cell enters G1-phase, remains there for many hours, and dies in G1-phase. Corresponds to Fig. 4c. Phase-contrast unavailable.

Supplementary video S12.

HT1080 FUCCI cell treated with 1μM selinexor that is born into G1-phase. Cell enters G1-phase (red), remains red for many hours, enters early S-phase (yellow), and dies. Corresponds to Fig. 4d. Phase-contrast unavailable.

Supplementary video S13.

HT1080 FUCCI cell treated with 1μM selinexor that is born into G1-phase. Cell enters G1-phase, remains red for many hours, transitions to S/G2-phase, and dies. Corresponds to Fig. 4e. Phase-contrast unavailable.

Supplementary video S14.

HT1080 FUCCI cell treated with 1μM selinexor that is born into G1-phase. After division, this cell enters G1-phase, and remains red and does not die.

Supplementary video S15.

HT1080 FUCCI cell treated with 1μM selinexor in G1-phase (red). Cell remains in G1-phase and dies after a long period of G1-phase.

Supplementary video S16.

HT1080 FUCCI cell treated with 1μM selinexor in G1-phase (red). Cell progresses to S/G2-phase and dies.

Supplementary video S17.

HT1080 FUCCI cell treated with 1μM selinexor in G1-phase (red). Cell remains red and does not die indicating arrest.

Supplementary video S18.

HT1080 FUCCI cell treated with 1μM selinexor in early S-phase (yellow). Cell progresses to S/G2-phase and dies.
